# Grooming Behavior as a Mechanism of Insect Disease Defense

**DOI:** 10.3390/insects4040609

**Published:** 2013-11-04

**Authors:** Marianna Zhukovskaya, Aya Yanagawa, Brian T. Forschler

**Affiliations:** 1 Sechenov Institute of Evolutionary Physiology and Biochemistry, Russian Academy of Scienses, Saint-Petersburg 194223, Russia; E-Mail: mzhukovskaya@yahoo.com; 2 Research Institute for Sustainable Humanosphere, Kyoto University, Uji 611-0011, Japan; 3 Department of Entomology, University of Georgia, Athens, GA 30602, USA; E-Mail: bfor@uga.edu

**Keywords:** grooming behavior, neural circuit, disease resistance, entomopathogen

## Abstract

Grooming is a well-recognized, multipurpose, behavior in arthropods and vertebrates. In this paper, we review the literature to highlight the physical function, neurophysiological mechanisms, and role that grooming plays in insect defense against pathogenic infection. The intricate relationships between the physical, neurological and immunological mechanisms of grooming are discussed to illustrate the importance of this behavior when examining the ecology of insect-pathogen interactions.

## 1. Introduction

In vertebrates, grooming has been described in terms of mutual expression of social acceptance and indicative of familial, as well as dominance, relationships between the different members of a group. Yet grooming is not always associated with social consequences as evidenced by the fact that the majority of animal species studied devote some time to grooming activities [1]. The integument has long been considered to be a mechanical barrier and the first line of defense against infection. A chitinous exoskeleton forms the integumentary boundary between an insect’s internal organs and the environment while also functioning in various other capacities including a platform for sensory and motor devices. This complex structure of epidermal origin contains numerous structural features that call for special maintenance to keep the cuticle in proper condition. The result is that insects like all terrestrial animals display various behaviors that are generally categorized as grooming [2]. Though the functions and associations are still ambiguous, insects of most orders also devote a lot of time in grooming activities. This review focuses on the functional role of grooming in insect disease defense as well as the neurological basis for grooming behaviors to highlight promising areas for future research.

As for hygiene behavior, special behavior directed toward the care of body surfaces is known from a wide range of animal species [3,4]. Earlier studies of grooming in insects were devoted to description, classification, and sequence of movements supposedly directed at the innate ‘need’ to keep clean [5,6,7]. A considerable amount of work has been devoted to specific groups of insects for example locusts that use the legs as a grooming device [8,9,10,11,12]. A wide variety of specialized structures used in cleaning the cuticle have been described in insects and is still the focus of considerable interest [7,13,14,15,16,17,18]. The importance and influence of grooming behavior as a topic of evolutionary theory is undeniable [1,17,19,20,21,22,23].

There are two broad categorizations used to describe grooming of the self or others. Autogrooming (self-grooming) is a classification that includes any act by the subject related to maintenance/care of the body surface and is considered an innate behavior represented across a plethora of vertebrate and invertebrate taxa [13,24]. Allogrooming (grooming another individual) is common in social vertebrates and eusocial insects, and occasionally observed in solitary insect species [25]. Despite the diversity of taxonomic groups involved, the main functions of grooming are amazingly similar, namely removal of foreign objects from the body surface, distribution of substances across the body surface and as a displacement behavior in stressful conditions [1,3,26,27]. Care of the body surface is thought to be important for disease prevention by elimination of pathogens, parasites and parasitoids [28,29,30,31,32]. Hydrophobic and bacteriostatic secretions spread over the body surface are known to improve the pathogen-barrier properties of the integument and also act as chemical signatures in both vertebrates and arthropods [28,30,33,34,35,36,37]. Irritants and mechanical stimulation often cause simple reflex reactions aimed at removing foreign material from the integument. Induced grooming in insects has been studied using dust particles, chemical irritants and weak mechanical stimulation [13,38,39,40,41,42]. In contrast, grooming also has been reported as an activity executed spontaneously without apparent external stimuli [5,43,44,45]. 

The inevitable ambiguity when attempting to classify a grooming behavior such as the simple scratching sweep that is often incorporated into a more complex sequence of defense-related behaviors. This is because of a high level of plasticity in grooming-related behaviors and often-subjective observations of the behavior itself. Thus exhibition of a behavioral reaction to a simple stimulus as evoking a scratching reflex make insects an excellent model for studying the neural circuitry underlying specific behaviors [46,47,48]. *Euphydryas phaeton* larvae regurgitate on attacking braconid parastoids, which react with prolonged grooming [49] while *Heliothis virescens* larvae use a head strike to apply an oral exudate to attacking *Cardiochiles nigriceps* females, which, in turn, groom [50,51]. Grooming behaviors have also been described as part of a diversity of more general behavioral routines such as the obligatory phase of host recognition [52], oviposition [53,54] and mating [55]. At a basic level grooming has been shown to eliminate extraneous amounts of the continuous efflux of cuticular hydrocarbons allowing proper operation of antennal sensilla [56]. Grooming does, however, come with an energy cost and is enhanced in satiated insects [57]. This review highlights insect grooming from the point of survival that is the protection from microbial infection.

## 2. Function of Grooming

Grooming in animals is a complex, multipurpose behavior as reflected in the “microstructure” of events used to phenotype such behaviors [23]. The cleaning of various body parts is generally organized in a particular sequence of observable behaviors, often with cephalo-caudal progression [1]. Insects employ two generalized strategies for grooming that are associated with the design of the mouthparts. One strategy is a deposition of debris onto a substrate as employed by members of the Orders Diptera and Lepidoptera that possess piercing-sucking, siphoning. The other is lapping mouthparts and ingestion as displayed by members of the Orthoptera and Coleoptera that have chewing mouthparts. Some insect’s combine both strategies, many hymenopterans, for example, groom their forelegs which possess a structure designed as an antennal cleaner with the mouthparts, while other body parts are rubbed against each other [17]. Another combined strategy involves honeybee allogrooming where the mouthparts are used to remove debris and parasitic mites from the body of a nestmate, but those items are not ingested [31,58]. Alternatively, the German cockroach possessing chewing mouthparts and achieves antennal debris removal through the scraping action that accompanies pulling the flagellum over the glossa and most of the debris is manipulated into the hypopharynx and ingested when grooming is completed [59].

Various functions have been proposed to explain insect grooming behavior: cleaning dust particles from sensory organs [60], smearing secreted or acquired cuticular lipids that constitute a familiar chemical fingerprint for insects [61,62], parasitoid disguise [63,64], collecting pollen particles as food [65] and removing ectoparasites or pathogens [65,66]. Grooming has been shown to assist with locomotion as wings and legs are groomed to clean and flatten scales (or feathers) to diminish air resistance during the flight [20,67] or tarsi are groomed to maintain adhesion of attachment pads [18]. The importance of grooming behavior for the maintenance of sensory organ acuity has been suggested for insects as diverse as crickets, cockroaches and flea beetles [52,59,68]. Recent data shows that eucalyptol, the general odorant that causes excitation of a receptor housed in male pheromone-sensitive antennal sensilla [69,70] induces pronounced changes in frequency and duration of cockroach antennal grooming. According to work with cockroaches, hydrophobic odorant molecules get adsorbed and dissolved by the hydrocarbons on the epidermal surface and should be removed to maintain high temporal resolution of odor signals [56].

**Figure 1:**
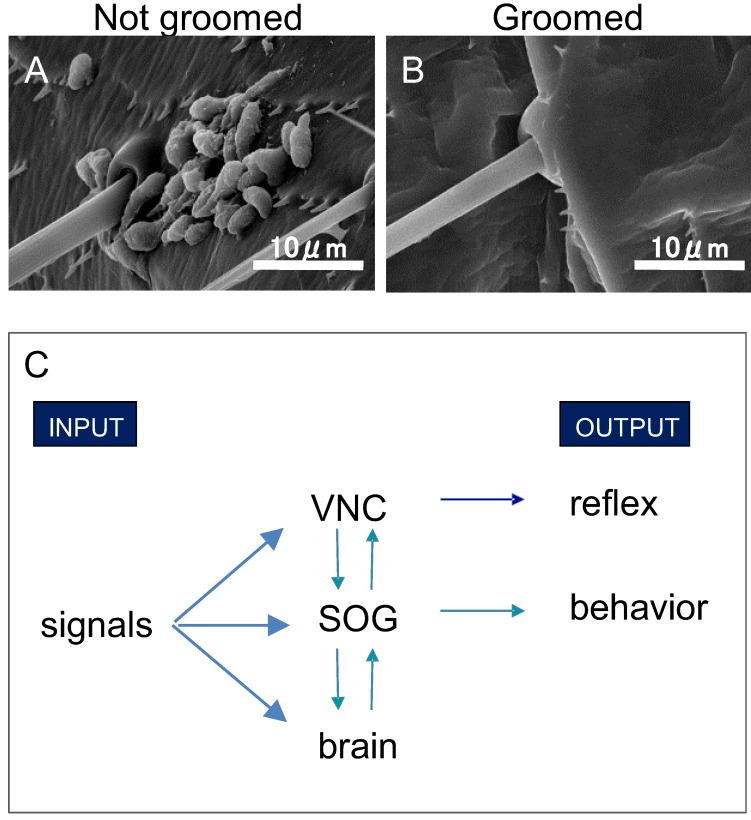
Scanning electron microscope images of nongroomed and groomed abdomen of *Coptotermes formosanus*. Pathogenic conidia completely cover the bases of sensilla (**A**), groomed abdomen have no conidium on their surface (**B**). (**C**) is the basic scheme of neural circuit of insect grooming. SOG is suboesophageal ganglion. VNC is ventral nerve cord.

Mammals and birds display displacement activities that have been classified as locomotory behaviors, cleaning behaviors (e.g., grooming) and manipulation of objects, which are often disrupted by a variety of stressors [71,72,73] similar phenomena have been detected in insects [26,27]. Exposure to novelty is a traditional research approach to displacement behavior in vertebrates as it causes abnormal patterns and interrupted bouts of grooming in rodents [4,23,74]. Insecticides can trigger a biochemical xenobiotic stress response, which is believed to share some pathways with stress responses caused by other menacing circumstances [75,76]. In insects, stress-response causes drastic changes of monoamines, such as octopamine, dopamine and tyramine, which in turn, can trigger behavioral changes [77,78,79,80]. It is possible that as part of their repertoire of behavioral stress-responses insects display displacement grooming [81,82,83,84,85,86]. Understanding insect grooming may provide insights into pesticide route of entry because the oral toxicity of substances that enhance grooming should increase in insects that include ingestion in their grooming routines while sucking insects would show a decrease in toxicity. For example, pyrethroid-resistant houseflies treated with fenvalerate (a pyretroid insecticide), immediately initiated a vigorous grooming behavior which allowed them to remove as much as 13% of the topically applied dose which led to insecticide transfer from fly bodies to the walls of holding vials [87]. Insecticide interactions also have highlighted the role of grooming in insect resistance to infections where exposure to low concentrations of insecticide resulted in greater infection in insects simultaneously exposed to entomopatogenic fungi [85] or nematodes [88]. Grooming is an especially important behavior in social insects where such contacts are believed to be integral in reducing horizontal transmission of disease [89,90] (Figure 1A,B). Anti-fungal, anti-microbial secretions spread by grooming also serve to reduce the probability that those microorganisms can damage the integrity of the insect cuticle for both social and solitary insects [28,30,37].

## 3. Neurobiology of Grooming

Mechanical or chemical stimulation causes movements aimed at eliminating foreign substances from the contaminated body surface [8,12,39,48,68,91,92,93]. It also has been shown that olfactory cues affect the pattern and frequency of grooming [94,95,96]. Irritant volatiles likely act through gustatory sensilla in flies, because *Phormia regina* subjected to an irritant vapor, evert the proboscis, regurgitate a droplet onto its tip (the labellum), and proceed to wipe the labellum against the substrate [97]. These data are in accordance with electrophysiological data, showing the response of contact chemosensilla to certain volatiles [98,99].Visual stimuli applied to only one eye cause an eye cleaning reflex [100], although the same behavior can be elicited by deflection of interommatidial mechanosensilla [68,91].

Aimed scratching, a stereotypic response to acute stimulation, has been used as a model for describing functional neuromorphology [48,92] and local control of leg movements [42,47,101]. Segmental circuits are integrated to produce complex intersegmental motor patterns [91]. In insects, the frontal ganglia (brain) is not required to produce simple aimed scratching, because the complete behaviour is executed even after transection of the connections anterior to the mesothoracic ganglion in locusts [47], and headless fly preparations [102,103]. Decapitated cockroaches are reported to groom their cercii and abdomen without special stimulation [104]. Simple scratching also has been used to describe as a process linked to non-associative learning such as habituation and dis-habituation of the cleaning reflex [105]. However not all neural circuitry related to behavior is under local control as spontaneous grooming was completely inhibited in headless flies [40]. Highly coordinated grooming, expressed by the complete repertoire of movements, is produced by the injection of parasitic wasp venom into the suboesophageal (SOG) ganglion of *Periplaneta americana* cockroaches [106,107]. SOG, which is placed below the brain and a part of central nervous system (CNS), generally controls insect movements. Segmental circuits can elicit simple sweeps that integrate into the greater intersegmental circuitry to produce higher order stereotypic actions, illustrative of neural plasticity that, at least in the cockroach, is controlled by the SOG dopaminergic network (Figure 1C). On the other hand, the absence of plasticity by feedback signals from sensilla was reported in the African praying mantis, *Sphodromantis lineola* that has mechanosensory sensilla in a femoral brush yet continue to perform eye cleaning movements after surgical removal of the brush [108]. In contrast, antennal grooming was more plastic and accomplished with a contralateral foreleg instead of the normally used ipsilateral foreleg in this species [108]. Our observations on antennal grooming in the American cockroach support these data, namely in the case of unsuccessful antennal cleaning, when after damage to the contralateral foretarsus prevent attempts to bring the antennal flagellum to the mouth subjects used both forelegs instead of the contralateral foreleg, often several times, to accomplish successful cleaning. 

There is little doubt that multiple neural feedback mechanisms tune grooming behavior according to current circumstances. In the German cockroach for example, the marginal sensilla located on the antennal basal segments 20–24 respond to the bending of flagellomeres and presumably play a role in determining the duration of flagellum grooming [109] while chemoreceptors at the base of the paraglossa participate in the ingestion of debris removed from the antennae during cleaning [59,110]. Grooming therefore is a part of the behavioral repertoire executed according to a hierarchy that produces an appropriate response to various stimuli affected by physiological conditions, such as satiation, arousal and aggression. It is believed that biogenic amines mediate these observed behaviors as treatment with dopamine (DA), octopamine (OA) and tyramine (TA) and their agonists and antagonists exert effects on grooming through different pathways: DA predominantly alters motor circuits [102,103,111], while OA and TA play a modulatory role [112] through general arousal/displacement mechanisms. 

## 4. Chemosensory Signatures

Insect behavior is often the result of a reaction to environmental signals hiding in the general background noise present in insect habitats [113]. This occurs by procedural knowledge processed from a neural circuitry that uses the difference between internally and externally generated signals to produce an appropriate behavior [114]. The importance of olfaction in host protection is highlighted by examples such as a flower that mimics olfactory and visual cues of fungi to avoid insect attack [115]. Herbivorous insects perceive the blend of odors from plants to discriminate host from non-host [116]. Blend-odor conceivably conveys essential signals also on pathogen perception in insects. Microbes vary with regard to a variety of measurable qualities such as competitive strength, attachment pattern, germination ability, and environmental adaptability [117], yet it is not clear what cues lead insects to recognize the presence of pathogens. Gripenberg *et al*. [118] suggested that insect species, which has a smaller host range, correlate with a narrower range of perceived odors. It is possible that the entomopathogen recognition process is analogous to the phytophagous insect host recognition system. Yet the importance of general odor reception as a cue for initiating behaviors should not be underestimated [119]. The simple fact that grooming enhances olfactory activity in cockroach supports the hypothesis of a critical role for grooming and entomopathogen recognition [56]. 

Insect olfaction seems to help protect insects from disease [120,121,122,123]. According to Pinho *et al.* [124], the volatiles of mushrooms could represent different groups of mushrooms at the species level and Mburu *et al.* [125] reported that high virulent entomopathogenic fungi shared similar volatiles. More studies are needed to clarify the interaction between odor-signal and insect perception. Integration of behavioral studies with the genetic and/or physiological pathways holds promise for a better understanding of the connection between behavior and disease resistance. The development of microRNA’s opens a new door for the study of grooming related behaviors that should be exploited given the potential of using insects in loss-of-function mutants for bioassay of behaviors [126,127,128].

The literature on animal behavior indicates that recognition of pathogens is a common trait across taxonomic categories. The nematode *Caenorhabditis* has been shown to detect specific chemical stimuli from bacterial pathogens [129], and display associative learning by avoiding indications of pathogenic bacteria using olfactory stimuli [130,131,132]. In vertebrates like rats, interactions between the immune system, sensory physiology and behavior can be affected by an immune response [133]. It is also well documented that vertebrates are capable of associative learning using taste aversive conditioning paradigms [134,135,136,137,138,139]. While, in insects, more studies are required to illustrate behavior as an integral strategy to cope with pathogens [66,140]. 

## 5. Detection of Pathogens

**Figure 2:**
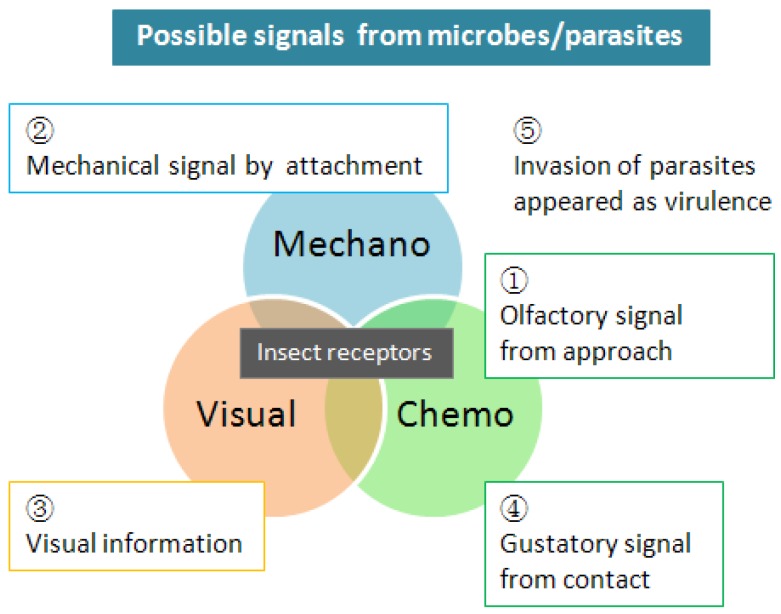
Microbes/parasites, which invade their host through insect cuticle are cleared due to grooming behavior. Olfaction will be the first signal from microbe [123,143,164], then physical [8,39] and gustatory [142,148,149] signals will be received by contact/attachments. Some hosts will be able to receive visual signals [31]. At the last stage, host insects probably find an invasion of pathogens by smell or behavior of infected individuals [163].

Insect perception of pathogens has long been thought to begin after contact [86,141,142]. Recently, however, numerous studies with social insects highlight the phenomenon of identifying pathogens prior to infection (Figure 2). Fouks *et al.* [143] reported that honey bees can detect parasite-contaminated flowers. Ants and honeybees demonstrate a communicated response to pathogens and groom more frequently in a contaminated environment in addition to grooming or removing infected larva [144,145,146,147]. *Formica podzolica* even display a response to their infected aphid mutualist partners [148]. 

The sensitivity of insects towards chemosensitive odors and tastes has been discussed in the context of food, sex or social interactions [149,150,151,152] but not in relation to disease. The hypothesis that chemoreception is involved in disease prevention may provide insights into how grooming behavior has evolved within different groups of insects [17], because it predicts that grooming should be related to selection pressure against certain pathogens in a given environment. It has been suggested that insects may have evolved specific chemoreceptors to detect pathogens [153,154,155]. *Drosophila* possess olfactory receptors that detect 1-octen-3-ol, a typical fungal odor [156,157], and it is not unreasonable to assume that the same selection pressure that preserves a host seeking behavior could also promote avoidance. A few papers examining full transcriptoms note that microbial infections are associated with changes in expression of olfactory-related genes like odorant binding proteins [158,159].

Termite hygiene behaviors are likely triggered by chemical information, because most termites are blind and therefore represent a good model system to test the hypothesis of chemoreceptive avoidance behavior. Several studies have reported that insects are repelled by the odor of strongly virulent entomopathogenic fungi [125,160,161,162]. Recent studies have revealed that termite antennae are sensitive to the odors of entomopathogenic fungi [95,163] this sensitivity varies according to the conditions of the bioassay [164]. Termites have been shown to avoid highly virulent *Metarhizium anisopliae* conidia more than less-virulent conidia [160,161]. It is possible that termites avoid aversive odors, not simply the most virulent pathogen; a categorization based on speed of mortality rather than overall pathogenic affects [165]. Future studies should examine more pathogens and fungal isolates that measure not just high virulence but factors such as decreased fecundity and mortality over an extended time frame [166]. 

There is still a long way to go to recognize behavior as an integral part of the strategies used by insects to cope with pathogens [66,140,167] and another area of exciting research is the initiation of humoral responses to pathogens [168,169].

## 6. Disease Prevention

**Figure 3:**
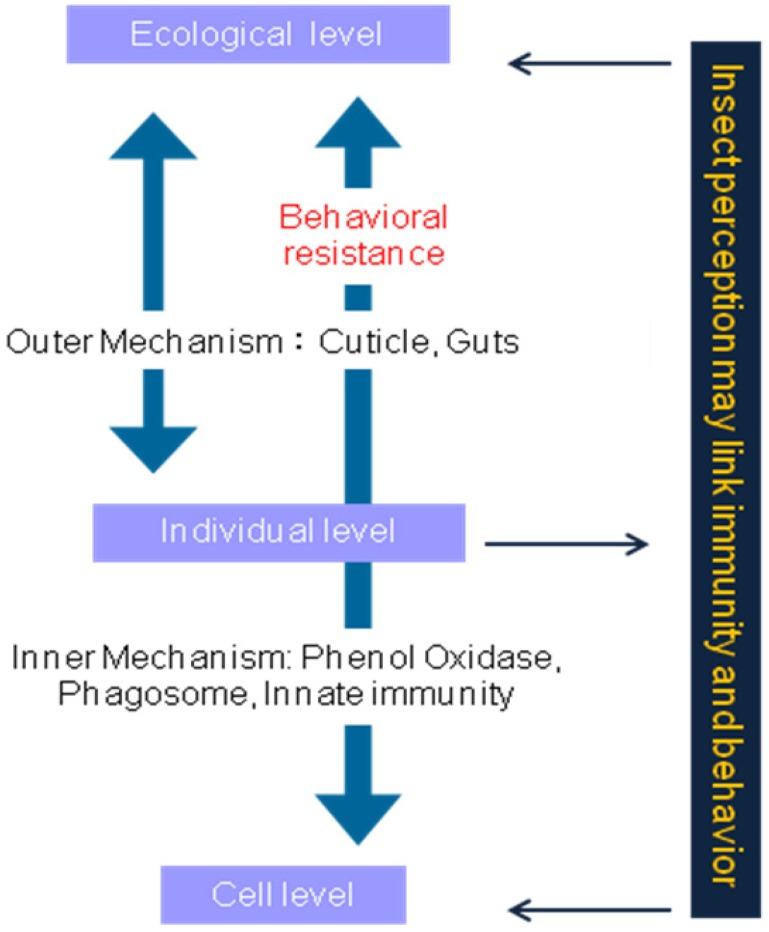
Interaction between Resistant Level Insects employing strategies to fight against microbial infection at cell, individual and ecological levels. Behavioral resistance is often the first defense in the infection stage.

Insect defenses against pathogens have been studied from the point of view of an overall immune response [140,142]. Vertebrates possess adaptive immunity yet debate continues on whether insect immunity has an adaptive component [170,171,172,173,174]. The known antimicrobial defense mechanisms include maintenance of physical barriers (epithelia), secretion of humoral mediators (antimicrobial peptides, reactive oxygen species), activation of proteolytic cascades leading to melanization and cellular functions including phagocytosis and encapsulation [142] (Figure 3). The regulation of antimicrobial peptide gene expression during systemic infection has shown that production of antimicrobial peptides by the fat body, analogous to the mammalian liver, are orchestrated through two signaling modules: the Toll and Imd pathways respond to microbial infection and lead to activation of NF-κB-like factors [142]. The strategies available to individual insects to withstand pathogen infections also have been viewed from the perspective of a cost/benefit trade-off [90,175]. Few studies have examined the role of behavior in insect disease defense [24,176,177,178]. However the role of insect grooming and hygienic activities is gaining recognition in the field of insect pathology [140].

Insects employ several strategies to fight back against microbial infection at cell, individual and ecological levels. Behavioral resistance will be one of the most initial defense in the infection stage.

Hygienic behavior has been shown to play a key role in disease prevention in insects [86,144,179]. It has also been documented that suppression of grooming behavior increases mortality in insect-pathogen bioassays [84,180,181,182,183]. Although grooming activities can be induced by aversive stimuli (mechanical or chemical), they also occur after oviposition or situations involving contact with potentially contaminated food, therefore there are few data supporting the specific hypothesis that grooming plays a role in the defense of insects against microbial infection [54,184,185,186,187].

Autogrooming can prevent infection from various microorganisms, especially in species like flies that live in an environment littered with bacteria, fungi and other microorganisms developing on decaying material [184,188]. Considering that many entomopathogenic fungi disperse as airborne conidia that penetrate the insect cuticle upon germination it is reasonable to hypothesize the same sensory neurons important in triggering grooming activities in response to dust particles [60] play a role in insect immunity to fungal infection. 

The trigger(s) that initiate insect behavioral reaction to pathogens is currently not well described. Increased grooming was noticed in the presence of parasitic nematodes for both earwigs and Japanese beetle larvae [189,190], however no data were collected to elucidate the sensory stimuli involved in such behavior. It is known that *Drosophila* spend a considerable amount of time grooming and that grooming systematically occurs after egg-laying [54,191]. Our unpublished observations also support the hypothesis that pathogen contact initiates grooming because most of the *Beauveria bassiana* conidia deposited on adult *Drosophila* are actively removed by grooming. There are reports that insect contact with pathogens can result in up/down regulation of gene expression, drive immune reaction and alter behavior [24,175,192]. In addition, toll-deficient *Drosophila* mutants show increased susceptibility to *B. bassiana* while infection leads to the expression of the antifungal peptide genes *Drosomycin* and *Metchnikowin* [193,194]. There are exciting opportunities to study the interaction between behavioral defense and humoral/cellular immune response in solitary insects and the trigger can be one of the most essential cues to clarify the associations. While, there are limited data in the literature on the question of whether insects respond to chemical or mechanical signals from microorganism by evoking grooming behavior.

Horizontal disease transmission can be significantly more serious in eusocial insects because colony members are closely related and frequently interact, often under conditions of high population densities [144,195,196,197]. The high risk of disease transmission within colonies is counterbalanced by cooperative behavioral defenses, often termed social immunity, that complement the immune response of individual group members [90,143,198]. It has often been reported that social insects such as ants [181,199], termites [29,177] and honeybees [65], help protect the colony from infection using allogrooming behaviors. Although a recent model suggests that allogrooming is less important than nest hygiene or immunity in social insect disease resistance [200].

Allogrooming is a well-known social behavior exhibited by numerous animal species that serves both hygienic and social functions [201]. Reduced allogrooming has been reported to be important in termite and ant social immunity in regard to removing pathogenic fungal conidia [29,36,85,159,202,203,204,205]. Allogrooming has also been implicated in the spread of glandular secretions or other antimicrobial substances such as the metapleural gland in ants [206,207,208,209,210,211,212] or gram-negative bacteria binding proteins in termites [169]. Ants have been shown to increase grooming of eggs and brood after exposure to pathogens [146]. In bees, autogrooming functions to remove external parasites such as *Varroa* mites [213] after which they conduct a ‘grooming dance’ that elicits allo-grooming from nestmates [31,65,214,215,216,217,218,219,220]. Social insects provide a compelling, comparative model for the study of grooming behavior in immunity and disease resistance.

## 7. Discussion and Conclusions

Maintenance of the cuticle, that physical barrier between an animal and the environment, can be considered the first line of defense against disease. The grooming behavior displayed by vertebrates and insects serve multiple functions, such as care and maintenance of the body surface and sensory organs. In insects mechano- and chemosensory sensitivity play an important role in the discovery of pathogenic organisms toward initiating grooming behaviors. Antimicrobial secretions spread by grooming provide insects with an additional role for this behavior. Sensory recognition can also trigger mobilization of immune response to compliment a suite of behavioral mechanisms, such as avoidance, self-medication and grooming [221]. Grooming behaviors vary by insect species and recognition of these behaviors is often not connected to disease defense although observations suggest that displacement grooming—well documented in mammals—also may be characteristic for stressed insects. The efficiency of grooming alone in protecting insects from disease is considered limited especially in solitary insects [222] yet there is ample evidence that the grooming component of social immunity is an effective tool in social insect disease defense [221]. 

Grooming behavior has long been observed and reported in many insects [13]. Future research should identify and classify these behaviors to aid in the systematic exploration of the physiological, neurological and pharmacologic basis of grooming. Occasional failures have been observed in biological control using microbial agents, which could be controlled by eliminating the agent with this behavior, but there are many other possible relevant factors. A better understanding of grooming should provide new insight toward the development of management practices using entomopathogens, leading to less damage to beneficial insects and consequently new possibilities for sustainable agricultural activity.
